# Clavulanic acid improves ethanol withdrawal symptoms in rats

**DOI:** 10.22038/ijbms.2020.39129.9287

**Published:** 2020-06

**Authors:** Ehsan Mohebbi, Mehdi Molavi, Mohammad Mohammadzadeh, Hossein Hosseinzadeh, Bahareh Amin

**Affiliations:** 1 Student Research Committee, Sabzevar University of Medical Sciences, Sabzevar, Iran; 2 Department of Internal Medicine, Sabzevar University of Medical Sciences, Mashhad, Iran; 3 Cellular and Molecular Research Center, Department of Physiology and Pharmacology, Faculty of Medicine, Sabzevar University of Medical Sciences, Sabzevar, Iran; 4 Department of Physiology and Pharmacology, Mashhad University of Medical Sciences, Mashhad, Iran; 5 Department of Pharmacodynamics and Toxicology, Pharmaceutical Research Center, School of Pharmacy, Mashhad University of Medical Sciences, Mashhad, Iran

**Keywords:** Alcohol withdrawal, Clavulanic acid, Elevated plus maze, Oxidative stress, Pentylenetetrazol, Rat

## Abstract

**Objective(s):**

Ethanol withdrawal following chronic use, is an important challenge clinically. In this study, the effect of clavulanic acid was evaluated on the symptoms of ethanol withdrawal in rats.

**Materials and Methods:**

Alcohol dependence was induced by the gavage of ethanol (10% v/v, 2 g/kg), twice daily for 10 days. Clavulanic acid (10, 20, 40, and 80 mg/kg) was administered concurrently with ethanol (sub-acute study), or a single dose after ethanol withdrawal (acute study). Six hours after the last dose of ethanol, anxiety was assessed by the elevated plus-maze (EPM). Seizure-like behavior was evaluated by a sub-convulsive dose of pentylenetetrazol (PTZ, 25 mg/kg/IP). Locomotor activity and motor coordination were measured by the open field and rotarod tests, respectively. Lipid peroxidation marker and antioxidant content were assessed through measuring malondialdehyde (MDA) and glutathione (GSH), respectively.

**Results:**

The number of entries and time spent on the open arms of EPM decreased during the withdrawal state. Motor coordination and locomotor activity were significantly decreased. In the sub-acute study, clavulanic acid 80 mg/kg increased time spent and the number of entries to the open arms of EPM, in withdrawn animals. Both motor incoordination and locomotor activity reduction were normalized by clavulanic acid (10, 20, 40 and 80 mg/kg). Withdrawal-induced PTZ kindling seizure was also suppressed by all of the doses. MDA increased, while GSH decreased after withdrawal. Clavulanic acid attenuated such changes.

**Conclusion:**

Clavulanic acid could prevent the development of alcohol withdrawal-induced anxiety and seizure. Alcohol withdrawal causes oxidative stress which can be prevented by clavulanic acid.

## Introduction

Alcohol is one of the most frequently abused substances throughout the world. Syndrome of alcohol withdrawal occurring after its chronic use is a fatal outcome and challenging to treat (1). Alcohol withdrawal consists of symptoms such as anxiety and decreased seizure threshold, which might be important reasons for the incompliance of abused people to give up this addiction. Benzodiazepines such as diazepam are currently prescribed as the first-line drugs in treatment of alcohol withdrawal, ameliorating both the anxiety and seizure. However, sedation and potential harm of dependence on them limit their use (2, 3). 

Although the neurobiological mechanisms underlying alcohol withdrawal are not fully understood, it was found that withdrawal of alcohol is associated with the elevated levels of oxidative stress parameters (4). Increased level of the excitatory amino acid, glutamate, mediating enhanced oxidative stress and subsequent neuronal death in different regions of the brain, also plays an important role (5-7). 

Clavulanic acid (CLAV) is an irreversible competitive inhibitor of bacterial beta-lactamase that naturally degrades and inactivates beta-lactam antibiotics. Therefore, it is commonly being used in combination with some beta-lactam antibiotics like amoxicillin and ticarcillin to prevent β-lactamase-mediated resistance. CLAV is structurally similar to beta-lactam antibiotics with no antibiotic effect (8). Furthermore, CLAV has a 2.5 fold greater penetrability to the CNS compared with β-lactam and ceftriaxone (9).

It has recently been reported that CLAV possesses neuroprotective effects (8), anti-seizure (10), anti-depressant, and anxiolytic properties (11). It has also shown stimulatory effect on the sexual behaviors (12), protective effect against neurodegenerative Parkinson’s and Alzheimer’s diseases (13), as well as protection against morphine’s tolerance, rewarding, and locomotor-sensitizing actions (14). 

Since susceptibility to seizure as well as anxiety are increased following withdrawal of ethanol abuse (15), in this study, we investigate whether CLAV could have any beneficial effects on the characteristic behaviors that occur after withdrawal of alcohol, in rats. Diazepam, a commonly prescribed benzodiazepine drug, was used as a positive control drug (16). Considering that oxidative stress has a key role in the events induced after alcohol withdrawal (7, 17), malondialdehyde (MDA), as a final product of polyunsaturated fatty acids peroxidation in the cells, and reduced glutathione (GSH), as an antioxidant modulator, were evaluated in the brains of animals after ethanol withdrawal.

## Materials and Methods

Diazepam was obtained from Darupakhsh Pharm Co, (Tehran, Iran). PTZ, MDA, and GSH were purchased from Sigma-Aldrich (St. Louis, MO). Ethanol was purchased from Merck, Germany. CLAV was a gift from Daana Pharmaceutical Co. (Tabriz, Iran). 

***Animals***

Adult male Wistar rats (210–250 g) were obtained from the animal center of School of Medicine, Sabzevar University of Medical Sciences, and housed in a quiet and temperature-controlled room (21±2 ^°^C), in which 12–12 hr light-dark cycle was maintained (08:00–20:00 hr light). All animals had free access to water and rat chow. The animal experimental protocol was approved by the ethical committee of Sabzevar University of Medical Sciences (medsab.rec.93.18) and was in accordance with internationally accepted Principles for Laboratory Animal Use and Care (18).

***Experimental procedure***

Ethanol (10% v/v) was given to animals as 2 g/kg (3 ml for each animal), via oral gavage, for 10 days (19). 

Ethanol solutions (10% v/v) were prepared from absolute ethanol and diluted in orange juice.

A sub-convulsive dose of pentylenetetrazol (PTZ, 25 mg/kg, IP), a GABA_A_ receptor antagonist was used to assess seizure susceptibility after withdrawal of chronic intermittent schedule of ethanol in rats (15). Anxiety behavior was evaluated by the elevated plus maze (EPM) apparatus.

Animals were divided into 18 groups (n=7 per group). 

Naïve groups: Rats received distilled water+orange juice for 10 days and were subjected to the open field, EPM and rotarod (group 1), or PTZ kindling (group 2) tests.

Control ethanol groups: Rats received distilled water+orange juice during exposure to ethanol and subjected to the open field, EPM and rotarod (group 3), or PTZ kindling (group 4) tests.

Sub-acute treated groups: Rats received CLAV (10, 20, 40, and 80 mg/kg) simultaneously with ethanol during 10 days, and subjected to the open field, EPM, and rotarod (groups 5-8) or PTZ kindling (groups 9-12) tests.

Positive control groups: Rats received diazepam (3 mg/kg), via oral gavage simultaneously with ethanol during 10 days, and subjected to the open field, EPM, and rotarod (group 13), or PTZ kindling tests (group 14).

CLAV groups: Rats received only CLAV 80 mg/kg for 10 days and subjected to the open field, EPM and rotarod tests (group 15).

Dz groups: Rats received only Dz 3 mg/kg for 10 days and subjected to the open field, EPM, and rotarod tests (group 16).

Acute treated group: Rats received an effective dose of CLAV in the sub-acute study, 6 hr after ethanol withdrawal, 30 min before testing, and subjected to the EPM or PTZ kindling tests (group 17).

Acute positive control group: Rats received diazepam (3 mg/kg), 6 hr after ethanol withdrawal, 30 min before testing, and subjected to the EPM or PTZ kindling tests (group 18).

***Behavioral tests***

Six hours after the last administration of ethanol, animals were sequentially subjected to the open field, EPM, and rotarod or PTZ kindling tests (20).

***Open field test***

The open-field box was a cubic chamber (60×60×40 cm), with a white floor divided into 16 equal squares. It was placed in a quiet room with dim light. Rats were delivered to the testing room, 30 min prior to the experiment. Each animal was gently placed in the center of the box and allowed to move freely over a 5-min period. The number of lines crossed by four paws in the open field apparatus was recorded. The apparatus was cleaned before each trial (21).

***Elevated plus maze (EPM)***

The EPM apparatus was constructed of two open (10 cm wide, 50 cm long with 5 cm wall sides) and two closed (10 cm wide, 50 cm long with 40 cm wall sides) arms, made of wood, arranged at 90 ^°^ angles (plus-shape) and intersected by a center platform (10×10 cm). The apparatus was raised 40 cm above the floor. Each animal was observed individually, for a 5-min test period. The total time spent on the open and closed arms was recorded. The maze was cleaned between tests (22).

***Rotarod test***

The rotarod test was performed to evaluate the motor coordination of animals, as previously described (23). Briefly, one day before the test, rats were pre-trained on the rotarod apparatus twice at 5 rpm speed for at least 5 min (BorjSanat, Iran), until they reached a stable baseline performance. On the day of the experiment, rats were evaluated on the rod rotating at an accelerating speed (from 4 to 40 rpm), over five min. The time taken for the falling of the rat (latency time) from the rotating rod, was recorded during the period of 1 min and the mean value of time (seconds on the rod) from the two or three testing trials was then calculated.

***PTZ kindling***

Animals in selected groups were injected a sub-convulsive dose of PTZ (25 mg/kg, IP). Each rat was separately placed into a Plexiglas box (30×40×40) for behavioral monitoring of convulsive episodes appearance, over a period of 30 min. Animals were observed in a successive order of the following scales of PTZ-induced convulsion (24). The percentage of animals showing any stages of convulsive behavior was measured after terminating alcohol administration.

Stage 0: no response;

Stage 1: ear and facial twitching;

Stage 2: myoclonic jerks without rearing;

Stage 3: myoclonic jerks, rearing;

Stage 4: turning over into side position, tonic-clonic seizures;

All tests were conducted in a quiet room with a low-intensity light 

***Measurement of MDA levels in the brain***

Peroxidation of lipids was estimated by measuring MDA as a lipid peroxidation end-product in cells. MDA in a reaction with reagent thiobarbituric acid (TBA) produces a pink colored complex (25). On the last day of study, the brains of animals were immediately dissected and stored at -70 ^°^C. Samples were weighed and mixed with 1.15% potassium chloride solution to prepare a homogenate (10%). After that, 0.5 ml of the yielded homogenate was added to 3 ml phosphoric acid (1%) and 1 ml TBA (0.6%) in a centrifuge tube and heated for 45 min in a boiling water bath. The product was left to cool and then 4 ml n-butanol was added, vortex-mixed for 1 min and centrifuged at 3000 rpm for 15 min. The organic layer was separated and absorbance was read at 532 nm. MDA was measured from a standard curve obtained from the commercially available standard MDA (Sigma-Aldrich). In the end, levels of MDA were expressed as nmol/gm tissue.

***Measurement of GSH levels in the brain***

Levels of GSH were estimated using reagent DTNB (2, 2´- dinitro-5, 5´-dithiodibenzoic acid), reacting with SH groups to produce a yellow complex. Briefly, a 10% tissue homogenate in buffer phosphate 7.4 was prepared and mixed with an equal volume of 10% trichloro acetic acid (TCA). After vortexing, the contents were centrifuged at 5000 rpm for 10 min, and supernatant (500 µl) was separated and mixed with a reaction mixture containing 2.5 ml 0.1 M phosphate buffer (pH 8.4) and 0.5 ml DTNB. Within 10 min, the color product absorbance was read at 412 nm using a spectrophotometer. GSH was determined from a standard curve made by commercially available standard GSH (Sigma-Aldrich). Levels of GSH were expressed as nmol/gm tissue (26).

***Data analysis***

Obtained results were analyzed by one-way ANOVA followed by Tukey’s *post hoc* tests, using SPSS version 13. Data were expressed as means ± SEM. For evaluating the percentage of animals convulsed, fisher’s exact test was used. The level of statistical significance was set at *P*<0.05.

## Results

***Sub-acute study***

*Open field test*

**Figure 1:**
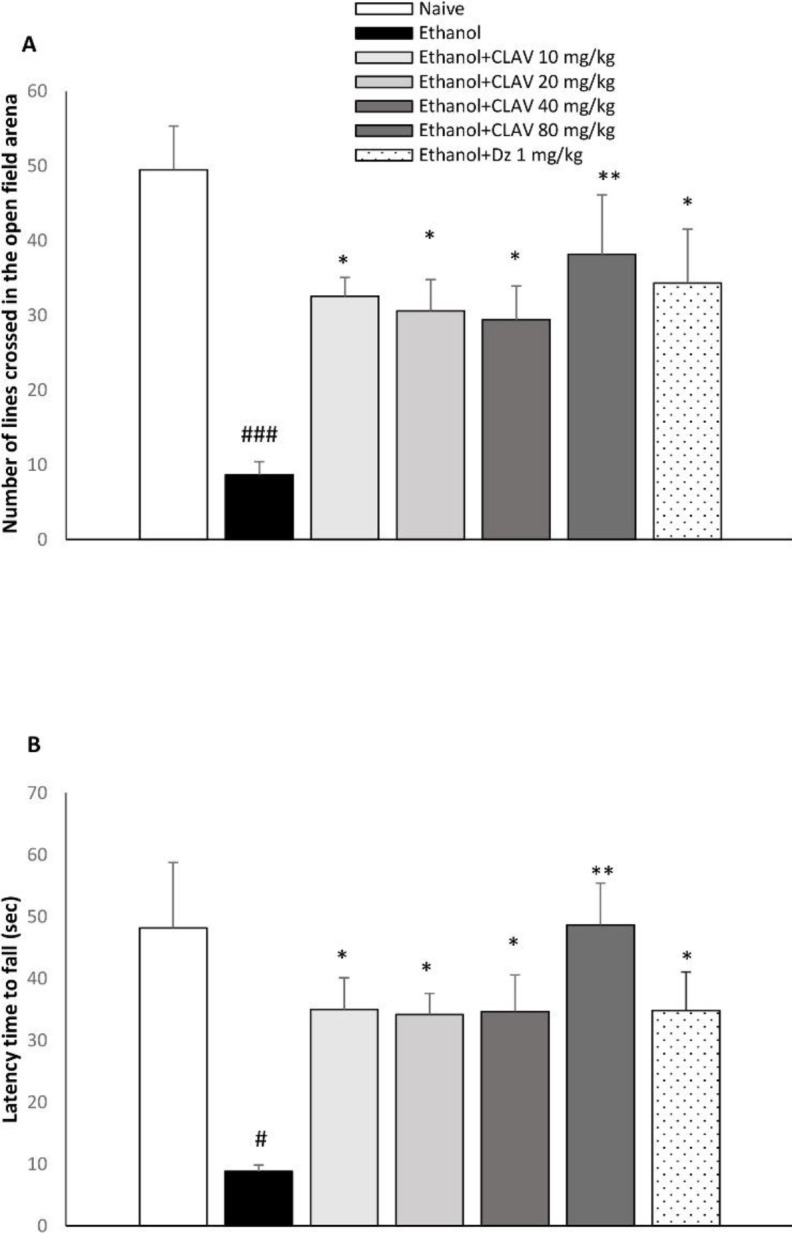
Effect of repeated gavage administration of clavulanic acid (CLAV: 10, 20, 40, and 80 mg/kg) on A: exploratory activity and B: latency time to fall from rotating rod, 6 hr after withdrawal from repeated administration of ethanol (10% v/v, 2 g/kg), twice a day for 10 days. Values are means ±SEM (n=7). Naïve (distilled water + orange juice) versus control ethanol group: # *P<*0.05, ### *P<*0.001. Statistical significant differences compared with ethanol control are indicated by * *P<*0.05, ** *P<*0.01. Tukey’s *post hoc* test, following one-way ANOVA, was used. Dz (diazepam, 3 mg/kg, via gavage) is the positive control group

Per se administration of diazepam or CLAV showed no significant variation on the number of lines crossed by animals compared with the naïve group, in the open field test. Rats abstinent from alcohol showed a significant reduction in the number of lines crossed in the open field apparatus as compared with naïve animals (F_6, 43_=8.3, *P*<0.001). Rats receiving CLAV at the doses of 10, 20, (*P*< 0.01), 40 (*P*<0.05), and 80 mg/kg (*P*<0.01), significantly improved exploratory activity as compared with respective animals treated with normal saline, over a 5-min period. The reference drug, diazepam (3 mg/kg), was also able to increase the exploratory activity of animals (*P*<0.01; Figure 1A).

***Rotarod test***

Per se administration of diazepam or CLAV showed no significant variation on the latency time compared with the naïve group, in the rotarod test. Statistical analysis of the rotarod test revealed that there was a significant effect (F _6, 41_= 4.9; *P*<0.001). Less time was spent on the rods of rotarod in the ethanol abstinent animals in the ethanol control group, compared with naïve animals (*P*<0.05). Conversely, locomotor activity was prolonged in withdrawn animals treated with CLAV at the doses of 10, 20, 40 (*P*<0.05), and 80 mg/kg (*P*<0.01), compared with the ethanol group. The performance of animals in staying on the revolving rod improved in animals treated with diazepam (3 mg/kg) as compared with ethanol groups (*P*<0.05; Figure 1B)‌.

***Elevated plus maze test***

**Figure 2:**
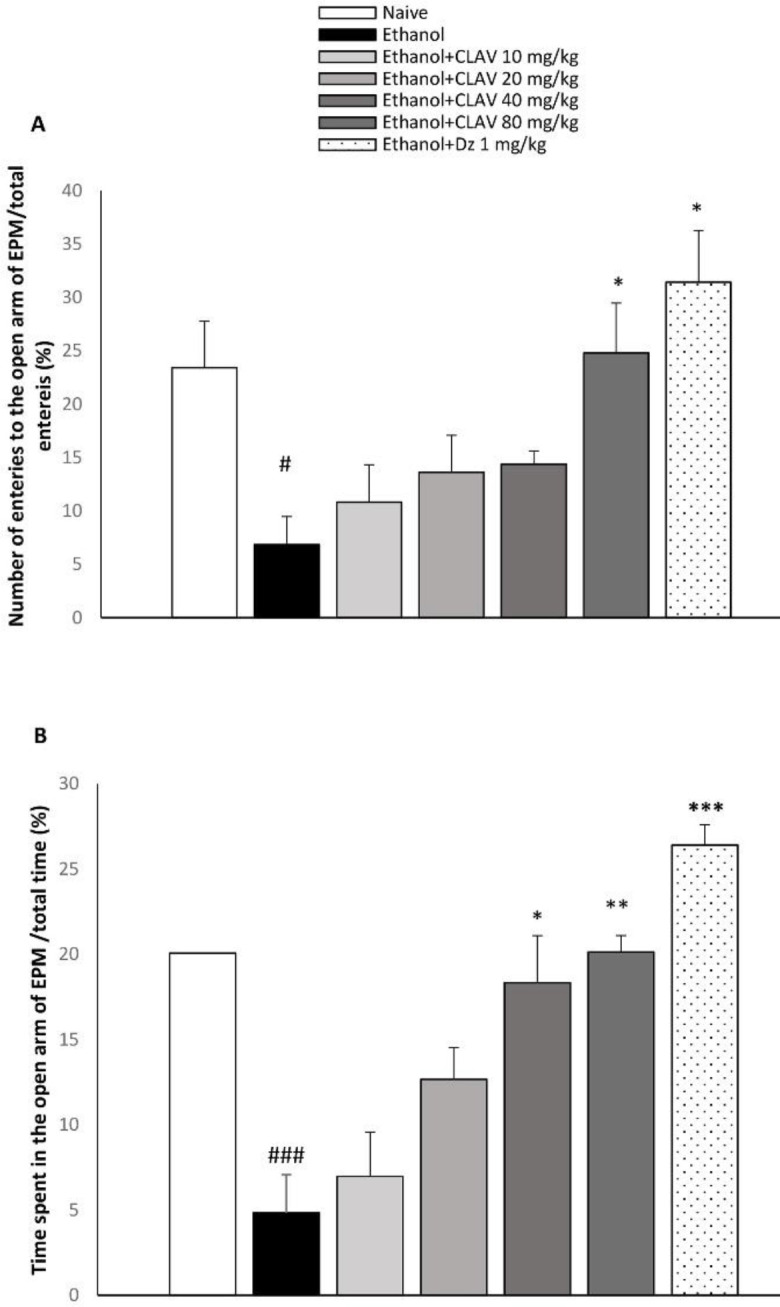
Effects of repeated gavage administration of clavulanic acid (CLAV: 10, 20, 40, and 80 mg/kg) on A: Percent of number of entries to the open arm/total entries and B: Percent of time spent in the open arm/total time in the elevated plus maze (EPM) in rats following withdrawal from repeated administration of ethanol (10% v/v, 2 g/kg), twice a day for 10 days. Values are means ±SEM. (n=8). Naïve (distilled water + orange juice) versus control ethanol group: # *P<*0.05, ### *P<*0.001. Statistical significant differences compared with ethanol controls are indicated by * *P<*0.05, ** *P<*0.01, and *** *P<*0.001. Tukey’s *post hoc* test, following one-way ANOVA, was used. Dz (diazepam, 3 mg/kg, via gavage) is the positive control group

By itself administration of diazepam or CLAV caused no significant variation on the number of entries or time percentage compared with the naïve group, in the rotarod test. Withdrawn alcoholic animals exhibited an enhanced avoidance of bright open spaces. Thus, the number of entries to the open arms (F_6, 44_=3.28; *P*<0.05) and time spent on the open arms decreased as compared with naïve animals (F _6, 44_= 4.172; *P*<0.001), (Figures 2A and B, respectively). Entries and time spent in the open arms of the EPM, were significantly higher in rats receiving CLAV (80 mg/kg), compared with those of the ethanol group (*P<0.*05, *P<0.*01, respectively), as did diazepam (3 mg/kg) (*P*<0.05, *P*<0.001) (Figures 2A and B, respectively). Time spent in the open arms of EPM increased by CLAV at 40 mg/kg (*P*<0.05) (Figure 2B).

***PTZ kindling test***

**Figure 3.**
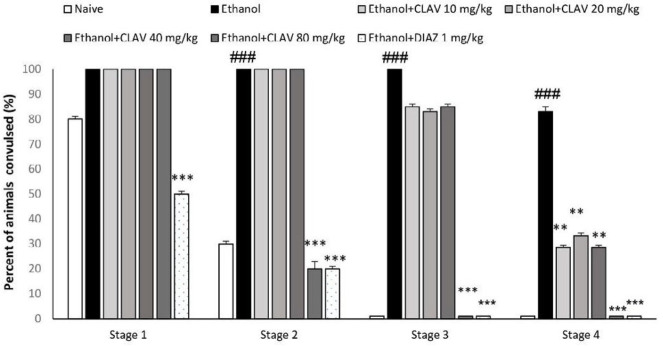
Effects of repeated gavage administration of clavulanic acid (CLAV: 10, 20, 40, and 80 mg/kg) on the susceptibility induced by sub-convulsive dose of penthyltetrazole (PTZ, 20 mg/kg, IP), following withdrawal from repeated administration of ethanol (10% v/v, 2 g/kg), twice a day for 10 days. Stage 1: ear and facial twitching; Stage 2: myoclonic jerks without rearing; Stage 3: myoclonic jerks and rearing; Stage 4: over into side position, tonic-clonic seizure. Values are means ±SEM for (n=8). Significant differences by Fisher’s exact test was used: Naïve versus control ethanol group: ### *P<*0.001. ** *P<*0.01, *** *P<*0.001, compared with ethanol. Dz (diazepam, 3 mg/kg, via gavage) is the positive control group

As indicated in Figure 3, almost all animals in the control ethanol group, showed four stages of convulsion (stages 1-4) in comparison to naïve animals following administration of PTZ, during the 30 min observation (*P*<0.001). CLAV at the doses of 10, 20, and 40 mg/kg was able to attenuate turning over into side position and tonic-clonic seizure (stage 4) (*P*<0.01). However, treating animals with a dose of 80 mg/kg of CLAV prevented myoclonic jerks without rearing (stage 2), myoclonic jerks and rearing (stage 3), and stage 4 (*P*<0.001). The group of animals receiving diazepam (3 mg/kg) also exhibited a significantly decreased percentage of convulsive behavior in all stages (*P*<0.001).

***Acute study***

The dose of 80 mg/kg of CLAV was chosen for the acute study. 

Values obtained in the PTZ kindling test in animals treated with a single dose of CLAV (80 mg/kg), 6 hr after ethanol withdrawal showed no significant difference from those of the ethanol control group (data not shown). Time spent in the open arm after withdrawal was increased in animals treated with a single dose of CLAV, 6 hr after ethanol abstinence, however, it didn’t show a significant effect (data not shown).

***Contents of MDA and GSH*** ***in animal brains***

**Figure 4:**
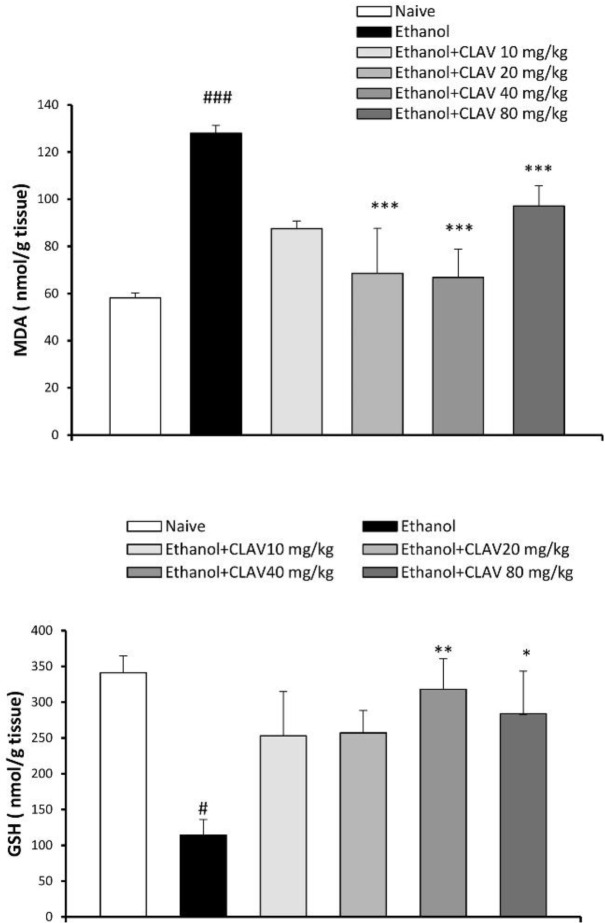
Effects of repeated gavage administration of clavulanic acid (CLAV: 10, 20, 40, and 80 mg/kg), on the brain contents of A: malondialdehyde (MDA) and B: glutathione (GSH) in rats following withdrawal from repeated administration of ethanol (10% v/v, 2 g/kg), twice a day for 10 days. Values are means±SEM (n=5). Naïve versus control ethanol group: # *P<*0.05, ### *P<*0.001. Statistical significant differences compared with ethanol are indicated by* *P<*0.05, ** *P<*0.01, and *** *P<*0.001. Tukey’s *post hoc* test following one-way ANOVA was used

Considering that absorbance is proportional to the concentration of MDA, abstinence from ethanol increased MDA content in the brain of the control group as compared with naïve animals (F 5, 27=23.8, *P*<0.001). In contrast, levels of GSH as an antioxidant factor that defeats oxidative stress attenuated (F 5, 29=4.13, *P*<0.05). Animals treated with CLAV (10, 20, 40, and 80 mg/kg) for 10 days, exhibited less MDA content (*P*<0.001, Figure 4A). 

GSH was higher in all groups receiving CLAV. However, it reached a significant level with 40 and 80 mg/kg of drug, compared with the ethanol group (*P*<0.01, *P*<0.05, respectively; Figure 4B). 

## Discussion

In the sub-acute study, withdrawal from repeated gavage of ethanol resulted in an anxiogenic-like effect as well as decreased motor activity in rats. Additionally, increased sensitivity to the sub-convulsive dose of PTZ was observed after withdrawal of ethanol. 

Gavage of CLAV concurrent with ethanol for 10 days, at all applied doses of 10, 20, 40, and 80 mg/kg increased threshold convulsion, exploratory activity, and motor performance in the abstinent ethanol group, as did the reference drug, diazepam. 

However, administration of CLAV only at the dose of 80 mg/kg, improved withdrawal-induced anxiety-like behavior. Anxiolytic activity of CLAV was previously reported by Kim *et al*., (2009) in the open field test on tamarins (11). 

One dose administration of CLAV was not able to significantly prevent behavioral symptoms that occur after alcohol withdrawal. This was in agreement with Gasior *et* *al*., (2012) reporting that acute administration of clavulanic acid (64 ng/kg to 5 mg/kg), failed to show anti-seizure activity in three models of seizure tests (27). It is speculated that repeated administration of CLAV is required for the attenuation of ethanol withdrawal-induced symptoms. 

MDA as a pro-oxidant factor increased in the brain homogenates of animals after withdrawal. In contrast, the antioxidant parameter, GSH decreased. It has been reported that oxidative parameters are elevated in the cerebrospinal fluid (CSF) of withdrawal patients (4, 5). Both increased MDA and decreased GSH after ethanol withdrawal were normalized in animals treated with CLAV.

Excitatory neurotransmitter, glutamate, plays a key role in the development of unwanted effects occurring after stopping alcohol consumption. Abstinence from ethanol and excessive extracellular glutamate lead to the formation of reactive oxygen species (ROS) that in turn cause damage to the nervous system, susceptibility to seizure, and anxiety (28). Up-regulation of the n-methyl-D aspartate (NMDA) receptors during chronic ethanol consumption may mediate the seizure associated with ethanol withdrawal (29). Targeting glutamate uptake and normalizing its concentration could be a promising option in preventing adverse effects after ethanol withdrawal in dependent animals (30). Beta-lactam, ceftriaxone alleviated alcohol withdrawal syndrome with the up-regulation of glutamate transporters and normalizing the concentration of glutamate (31). In a recent study, clavulanic acid decreased the reinforcing efficacy of cocaine by enhancing glutamate transporter 1 (GLT1), which reuptakes the majority of extracellular glutamate (32). 

Neuroprotective effects of CLAV have been reported in previous studies. As reported by Kost *et al*., (2012), CLAV prevented damage induced by neurotoxin 1-methyl-4-phenylpyridinium (MPP+), a model of Parkinson’s disease via decreasing the levels of mitochondrial-mediated cell death pathway including Bax, cytochrome C, as well as caspases 3 and 9. While Anti-apoptotic Bcl-xl levels were returned to normal levels (33). 

Modifying the serotonin system might be another mechanism by which CLAV showed antianxiety and protection against PTZ kindling. In some studies, the role of serotonin has been demonstrated on seizure induction, as well as anxiogenic behaviors, during ethanol withdrawal (34, 35).

 CLAV stimulated sexual behavior as did selective serotonin receptor inhibitor (SSRI), paroxetine. 

Considering that this effect was reduced by mianserin, a serotonin 5HT(2c) receptor antagonist, an increase of central serotonin neurotransmission is also likely to participate in the effects of clavulanic acid (12). According to a previous report, serotonin syndrome was observed after a single dose of co-amoxiclav (clavulanic acid+amoxicillin) during treatment with venlafaxine (36). 

Benzodiazepines have been the gold standard drugs during discontinuation state of ethanol. With potentiating gamma amino butyric acid (GABA_A_) receptor signaling, benzodiazepines struggle with the excessive glutamate-induced hyperexcitation during the cessation of ethanol. Although in our study motor performance and exploration of animals did not alter with 3 mg/kg of diazepam, these drugs are associated with many adverse effects such as sedation, tolerance, and dependence (37). However, such adverse effects have not been reported with CLAV, which could be an advantage of this drug.

We selected gavage drug administration, which is the most preferred, safe, and readily available route of drug prescription with highest patient compliance (38).

Like ceftriaxone, presence of the β-lactam ring is further supported in the neuroprotective effect observed with clavulanic acid. However, better bioavailability and permeability to CNS of clavulanic acid (9), makes it a good promise in the treatment of ethanol withdrawal. 

## Conclusion

We found that that clavulanic acid via the oral route has the potential to attenuate unwanted harmful symptoms, such as anxiety and seizure vulnerability induced following ethanol withdrawal. However, the anti-anxiety effect is exerted at the higher doses. The antioxidant effect is at least in part responsible for the observed neuroprotective effects of clavulanic acid. Further studies are required to clarify the exact mechanisms that contribute to decreasing the signs and symptoms of ethanol withdrawal. 
